# Mechanisms underlying abnormalities of immune activation/coagulation in HIV infection

**DOI:** 10.7448/IAS.17.4.19477

**Published:** 2014-11-02

**Authors:** Clifford Lane

**Affiliations:** Clinical and Molecular Retrovirology Section, National Institute of Allergy and Infectious Diseases, National Institute of Health, Bethesda, MD, USA

## Abstract

Immune activation has been recognized as an important component of the pathogenesis of HIV infection since the first recognition of cases of AIDS in the early 1980s. Early in the AIDS epidemic, patients with HIV infection were noted to have elevated levels of serum immunoglobulins. CD38 expression on CD4+ T cells was shown to be an independent predictor of survival in 1999. The characterization of HIV-associated immune activation has become progressively sophisticated over the past several years. A consistent finding has been an association of poor clinical outcomes with markers of monocyte activation (IL-6 and sCD14) and/or coagulation (D-dimer). These relationships have been shown to exist even in patients with plasma levels of HIV-1<50 copies/ml. While it is generally accepted that immune activation is related to HIV infection, there is less clarity regarding the pathways that lead to its expression. Among the forces reported to drive HIV-associated immune activation are innate and adaptive immune responses to HIV and related co-infections, homeostatic responses to CD4+ T cell depletion and translocation of microbial products across the intestinal wall. Recent work has identified a potential role for “defective” HIV-1 transcripts in driving immune activation. Studies examining the connections between the adaptive immune system and the coagulation cascade have led to the identification of PAR-1 as a potential target for therapeutic intervention. Despite the successes experienced with cART, persistent immune activation in association with HIV infection remains a scientific and clinical problem that is yet to be solved.

